# Direction-Selective Circuitry in Rat Retina Develops Independently of GABAergic, Cholinergic and Action Potential Activity

**DOI:** 10.1371/journal.pone.0019477

**Published:** 2011-05-05

**Authors:** Le Sun, Xu Han, Shigang He

**Affiliations:** State Key Laboratory of Brain and Cognitive Sciences, Institute of Biophysics, Chinese Academy of Sciences, Beijing, China; INSERM U901, France

## Abstract

The ON-OFF direction selective ganglion cells (DSGCs) in the mammalian retina code image motion by responding much more strongly to movement in one direction. They do so by receiving inhibitory inputs selectively from a particular sector of processes of the overlapping starburst amacrine cells, a type of retinal interneuron. The mechanisms of establishment and regulation of this selective connection are unknown. Here, we report that in the rat retina, the morphology, physiology of the ON-OFF DSGCs and the circuitry for coding motion directions develop normally with pharmacological blockade of GABAergic, cholinergic activity and/or action potentials for over two weeks from birth. With recent results demonstrating light independent formation of the retinal DS circuitry, our results strongly suggest the formation of the circuitry, i.e., the connections between the second and third order neurons in the visual system, can be genetically programmed, although emergence of direction selectivity in the visual cortex appears to require visual experience.

## Introduction

Image motion is an important survival cue to animals. Among fewer than 20 types of retinal ganglion cells (RGCs) in the mouse [1], three are devoted to coding motion directions [2], [3], [4]. The ON-OFF direction selective ganglion cells (DSGCs) respond strongest when images move in a particular preferred direction, but very weakly when they move in the opposite null direction [5]. The mechanism for direction selectivity (DS) is a spatially asymmetrical inhibition [6] that DSGCs only receive inhibitory inputs from starburst amacrine cells (SACs) spatially offsetting to the direction in which image motion induces very little or no response, providing a physical entity for the asymmetrical inhibition [7]. The output of SACs is direction selective (DS) [8], produced cell-autonomously [9] or by reciprocal inhibition between overlapping SAC processes [10]. It is not known how the selective connections are formed between dendrites of DSGCs and the processes of SACs extending centrifugally in coincidental null directions. Recent findings have ruled out involvement of light driven activities and bipolar cell outputs [11], [12], [13].

SAC contains both acetylcholine (ACh) and GABA [14], [15], and GABA plays a critical role in the computation of motion direction: blocking GABA receptors abolished DS [16] and the asymmetrical responses in its varicosities are mediated by reciprocal GABA inhibition [10], in addition to cell autonomous properties [9]. The enzyme synthesizing GABA is present very early in development [17], when the synchronous activities are modulated by GABA and glycine [18]. Activating GABA receptor induces an inward current [19], [20] that becomes outward around postnatal day 6 (P6) with the expression of the potassium-chloride cotransporter, KCC2 [21], [22]. It has been shown recently that direction selective circuitry develops normally after blocking GABA transmission in the 2^nd^ week of development [23]. It is not clear if activation of GABA receptors is necessary for the initial formation of contacts between SACs and DSGCs.

Spontaneous, synchronized propagating activities in the early developing retina [24], simultaneously activating RGCs and their target neurons, have been suggested to play a role in the formation of eye specific lamination in the lateral geniculate nucleus [25], [26], [27], and the precise geniculocortical mapping [28], [29], [30], although controversial evidence existed [31]. During this period, SACs express voltage gated sodium channels [32] and are activated synchronously with RGCs by propagating activities [33], it is conceivable that the wave-induced synchronous activities provide a possibility for the DSGCs and SACs to form selective connections through a spike timing dependent plasticity (STDP) [34], a Hebbian mechanism. From postnatal day 0 (P0) to P11, ACh release [35], [36] triggered by pace-making activity of SACs [37] is critical for propagating activities. Normal DS circuitry has been observed in the nicotinic ACh receptor β2 subunit deficient mice in which retinal synchronous activities were diminished [13], arguing that synchronized activities are not important. A recent paper showed increased frequency of synchronous activity in the same mouse line [38] and therefore, strengthened STDP.

In this study, we used intraocular injection of pharmacological reagents to block the GABAergic activity, ACh driven synchronous activity or/and Na^+^ dependent spiking activity, and demonstrated the morphology, physiology and the DS circuitry develop independently of GABAergic, cholinergic or spiking activity. Our findings strongly suggest a genetic mechanism in this circuitry formation.

## Results

DSGCs are observed in the rat retina as early as P13, showing very similar dendritic morphology and physiology to their counterparts documented in the rabbit and mouse (Fig. S1), confirming that DS was present in the rat retina at the onset of the light sensitivity, similar to what was reported in the mouse [12].

### Blocking GABA_A_ receptors does not affect the development of DS circuitry

**Figure 1:**
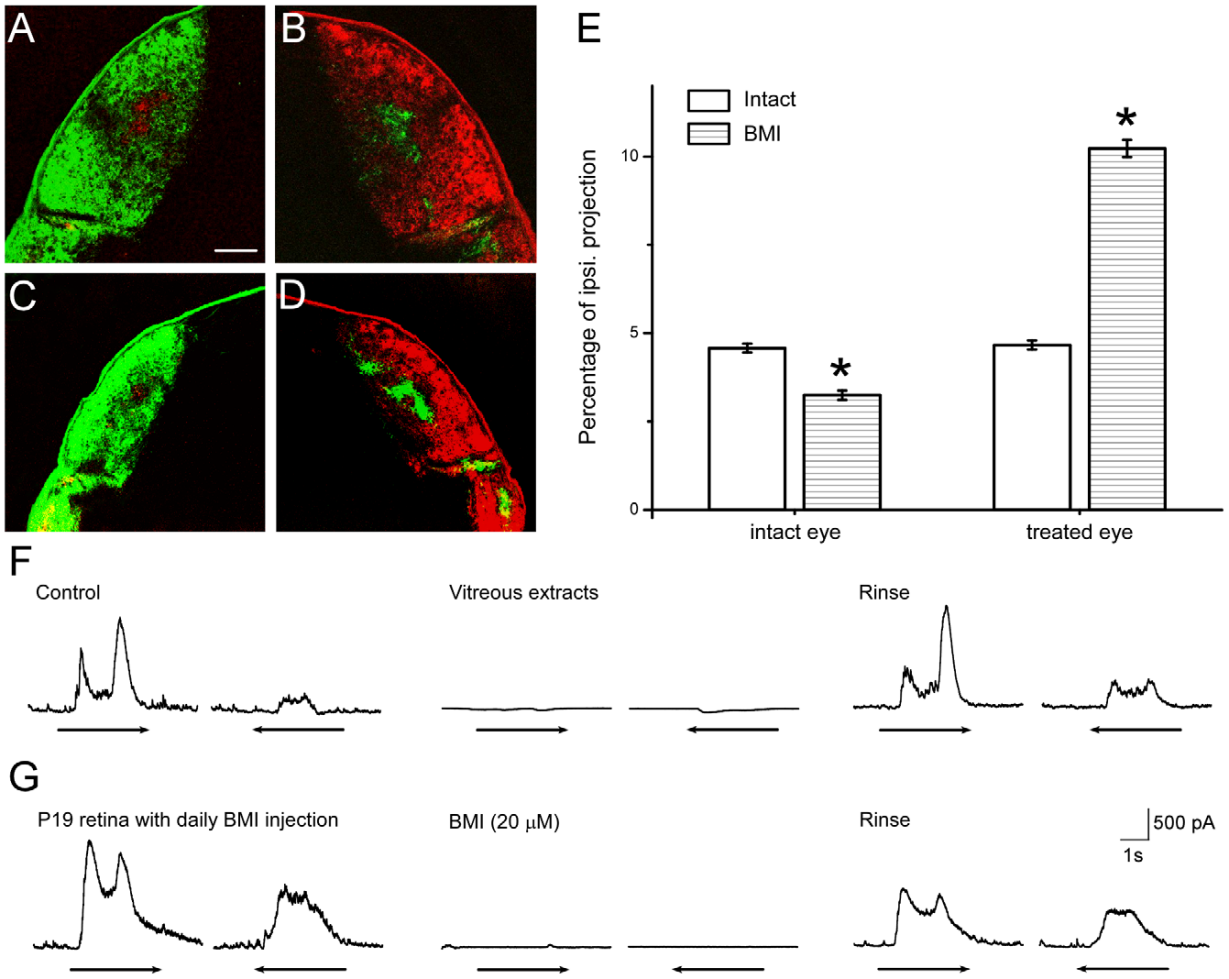
Daily BMI injection effectively blocked GABAergic activities. A, B: Retino-geniculate projection in control animal visualized by intraocular injection of CTB. C, D: Disrupted retino-geniculate projection showing much expanded ipsilateral and contralateral projection of the injected eye (green) and clearly reduced innervations of the uninjected eye (red). Scale bar: 200 µm. E: Comparison of the ipsilateral projection between intact and treated eye. N = 10, Data were analyzed using one way ANOVA, and presented in AVG±SE. *: p<0.05. The BMI group received monocular BMI injection. F: Vitreous extracts from an eye injected 24 h earlier completely blocked the inhibitory inputs of a DSGC in a control retina, showing lasting effectiveness of the BMI. G: In a retina receiving 19 consecutive BMI injections, the inhibitory inputs to a DSGC were blocked by 20 µM BMI, showing no tolerance was induced by repeated injection.

We first tested if GABA activities play any role in the formation of retinal DS circuitry by daily intravitreous injection of a potent GABA_A_ receptor blocker, bicuculline methiodide (BMI) [39], from birth to the day before experiment (P15–18). We verified the effectiveness of daily injection from three aspects. First, retinal geniculate projection was severely disrupted, showing expanded contra- and ipsi-projection of the injected eye (Fig. 1A–E), presumably due to increased activities induced by GABA blockade. Second, vitreous extracts from one eye injected 24 h earlier, completely blocked inhibitory inputs to a DSGC in another retina despite the dilution in the perfusion chamber (Fig. 1F), indicating the BMI effect lasted at least for 24 h, sufficiently long lasting for our daily injections. Third, low dose BMI completely blocked inhibitory input in a retina receiving 19 consecutive injections from birth (Fig. 1G), demonstrating no tolerance to repeated injections.

**Figure 2:**
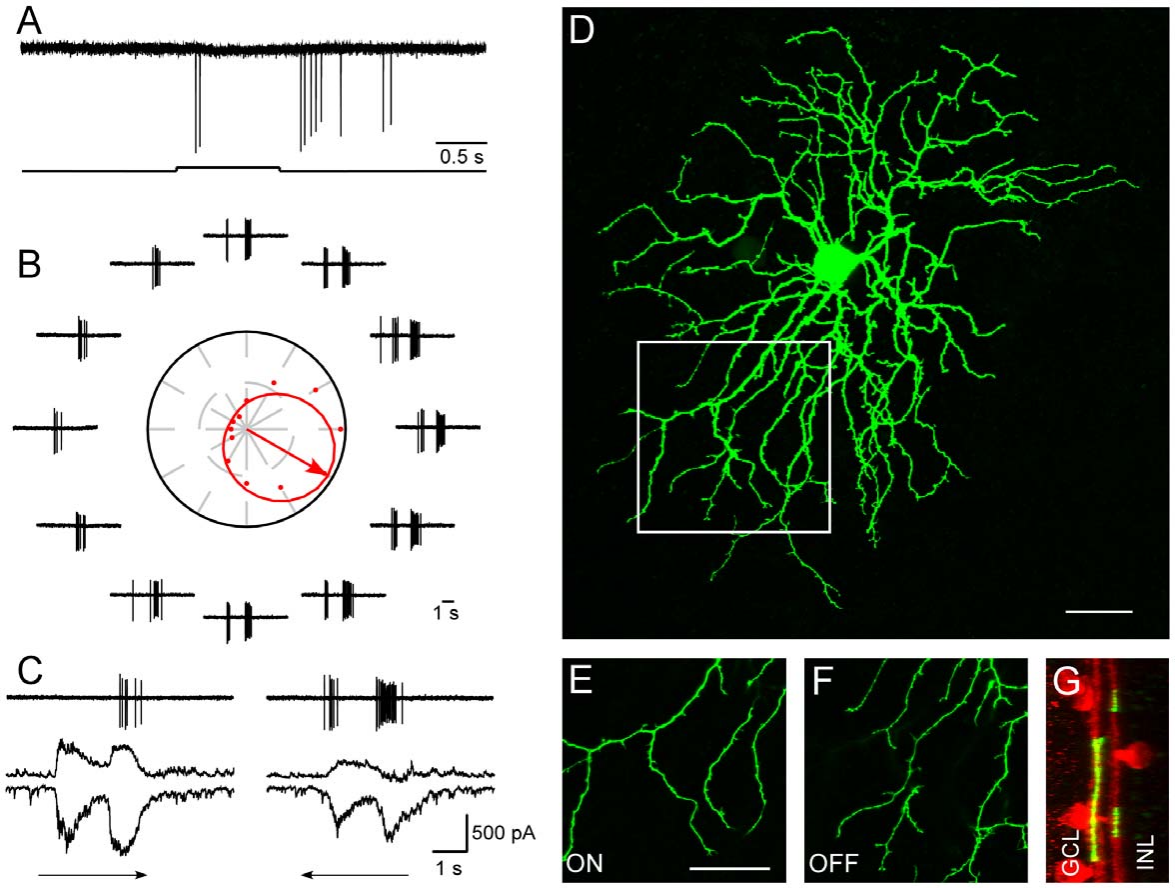
Normal DSGCs can be found in retinas receiving 16 to 19 daily BMI injections. A: A DSGC responded to the onset and termination of a stationary flashing spot. B: Directional responses to a rectangle moving in 12 directions. The preferred direction is represented by the arrow in the middle of polar plot surrounded by traces of action potentials. C: Asymmetrical synaptic inputs recorded when membrane potential was held at −65 mV and 0 mV, respectively. Much larger inhibitory inputs were observed when visual stimulus was moving in the null direction. D: Dendritic morphology of the recorded cell. A section of the dendritic field was enlarged showing clear ON (E) and OFF (F) segregation. Tight costratification with cholinergic plexus was clear (G). Scale bar: 30 µm.

**Figure 3:**
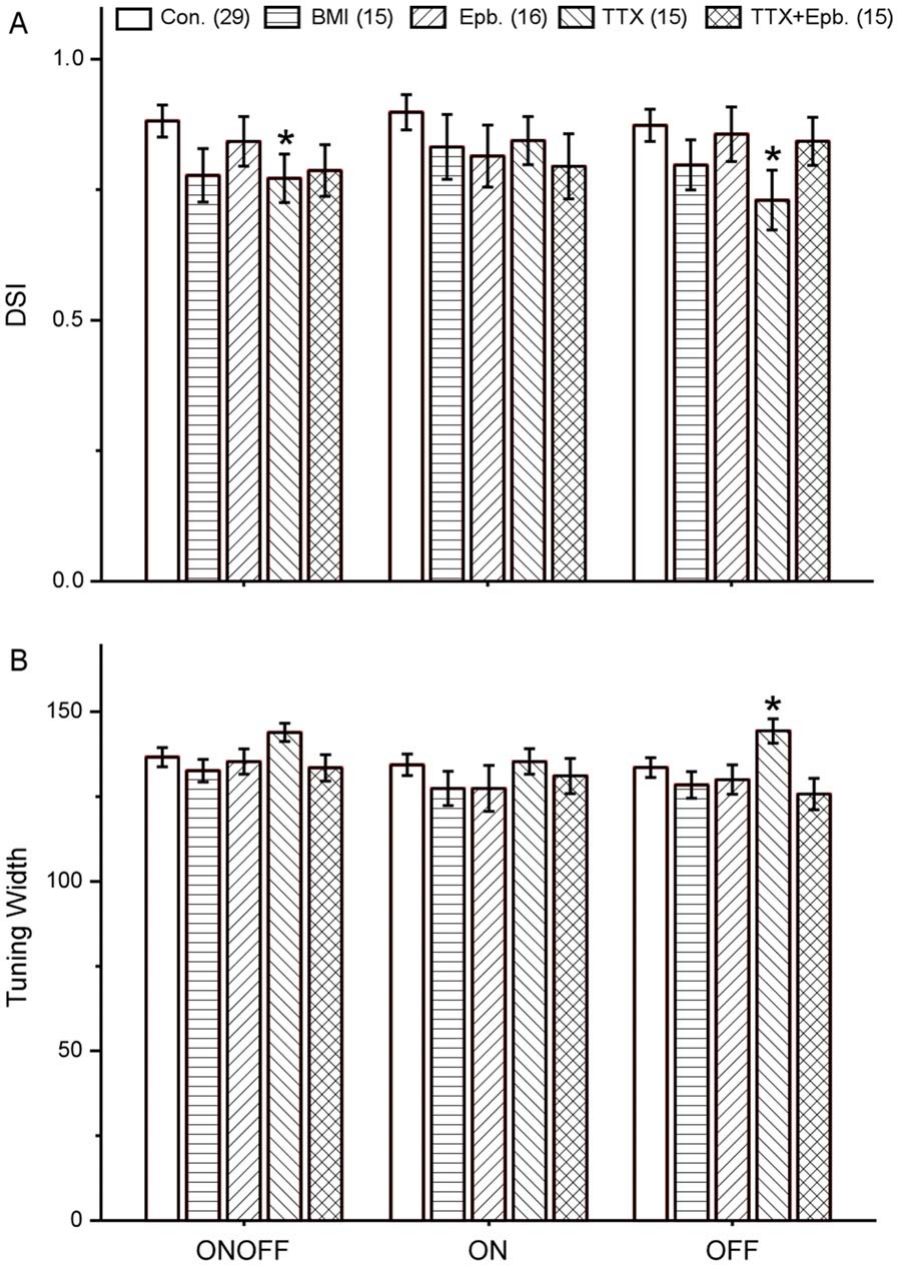
Comparison of DSI and turning width. Population data comparing direction selective index (A) and the width of direction tuning curves (B) of DSGCs in control retina and retinas treated with BMI, Epb, TTX and the mixture of Epb and TTX. Data were analyzed using one way ANOVA, and presented in AVG±SE. *: p<0.05. Cell number is described in parentheses.

DSGCs with normal light responses, direction selectivity and dendritic morphology could be found in retinas receiving 16 to 19 consecutive injections. Identified DSGCs responded to the onset and termination of a stationary flashing spot (Fig. 2A). To a moving rectangle, clear DS responses could be observed (Fig. 2B) with asymmetrical excitatory and inhibitory synaptic inputs (Fig. 2C). Bistratified dendritic morphology is illustrated in Fig. 2D, a section enlarged in 2E and F showing clear ramification in the ON and OFF sublaminae. Dendrites tightly costratified with cholinergic bands (Fig. 2G) in the inner plexiform layer (IPL). All together, fifteen DSGCs were recorded from ten BMI treated retinas. Data were summarized in Fig. 3. These experiments demonstrated that retinal DS circuitry develop normally without GABA mediated activities.

**Figure 4:**
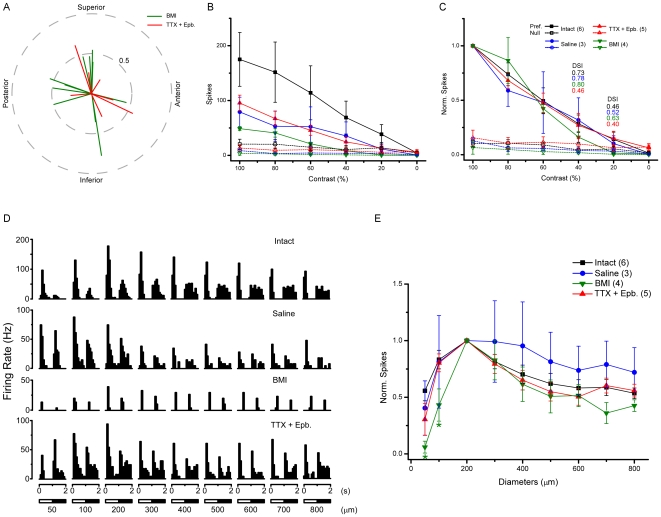
Homeostatic properties of DSGCs in BMI and Epb plus TTX treated retinas. A: DSGCs with preferred direction falling into all four subtypes were observed. The length of the lines represents normalized response magnitude (vector sum divided by total number of spikes), the angle preferred direction. B: Clear directional responses to square wave gratings with contrast as low as 20%, normalized data are shown in C (numbers in corresponding color represent DSI of treated DSGCs). D: Responses to a stationary flashing spot of different size. Although cells in treated retina generally exhibit lower firing rate, clear center-surround interaction can be seen especially in normalized data in E. Black: Intact; Blue: Saline; Green: BMI; Red: Epb+TTX. Numbers in parentheses indicate the cell number.

All four subtypes of DSGCs [40], [41] were detected in BMI treated retinas (Fig. 4A). They were quite directional to drifting gratings with a contrast as low as 20% (Fig. 4B, C), although DSGCs in the treated retinas generally yielded weaker responses (Fig. 4B). The center-surround interaction was also intact as shown in histograms (Fig. 4D) and in normalized area-response curve (Fig. 4E), responses increased with the size of spot initially and decreased when the spot entered the inhibitory surround.

### Retinal DS circuitry develops independently of ACh induced synchronized activity

We then tested the roles of synchronized, ACh driven activity in the formation of retinal DS circuitry. We performed 14 to 19 consecutive daily intravitreous injections of a potent cholinergic agonist, epibatidine (Epb) which completely blocked synchronized activities with bath application [25], [26], [27], [29]. Similar control experiments confirmed the sustaining effectiveness of Epb: 1) the retino-geniculate projection showed severe disruption with the ipsilateral projection of the treated eye to the LGN almost disappeared, and the ipsilateral axon terminals of the uninjected eye occupied a larger and diffused region (Fig. S2C–E). 2) Twenty four hours after an injection, the vitreous extracts from an injected eye completely blocked synchronized firing of a pair of neighboring RGCs of another early postnatal retina (P7), suggesting the intraocular concentration was sufficient to block synchronized activities throughout the treatment (Fig. S2F). 3) Bath application of 40 nM Epb blocked synchronous bursting of a pair of neighboring RGCs in a P9 retina receiving 9 consecutive intraocular injections, indicating no tolerance to Epb was caused by the extended treatment (Fig. S2G).

**Figure 5:**
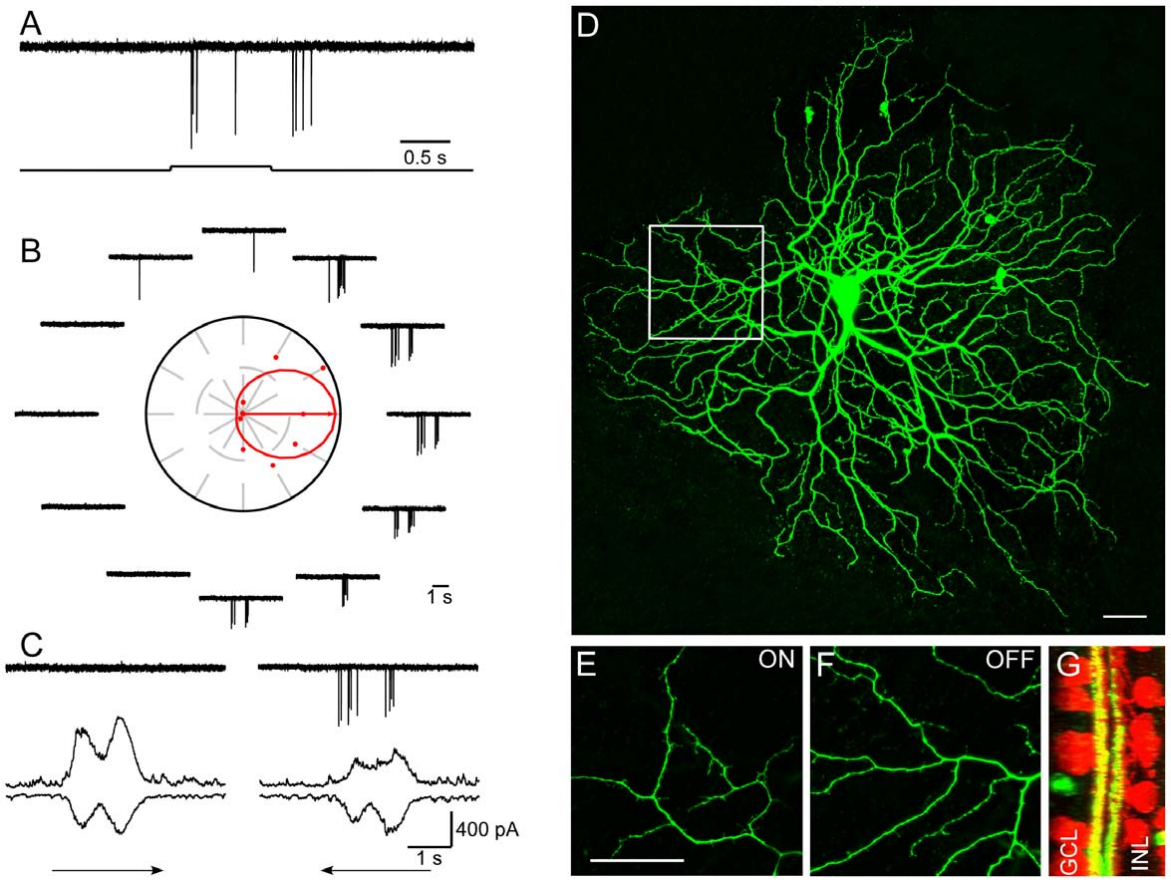
A DSGC in the retina treated with Epb for 15 days. A: To a stationary flashing spot, the cell responded to both the onset and termination. B: To a rectangle moving in 12 directions, highly asymmetrical responses could be observed. Polar plot shown in the middle of response traces with the vector sum represented by the arrow and the tuning curve by the circle. C: Excitatory and inhibitory synaptic inputs recorded when membrane potential was held at −65 mV and 0 mV, respectively. D: Dendritic morphology of the recorded cell. E–G: High magnification of the region indicated by the white square in D. E and F: Showing dendrites in the ON and OFF sublamina, respectively. G: Side view, showing costratification with ChAT bands. Scale bar: 30 µm.

Retinal DS was still intact when patch clamp recordings were made from retinas receiving daily Epb injection. DSGCs exhibited responses to the onset and termination of a flashing spot (Fig. 5A), clearly directional responses to a moving rectangle (Fig. 5B), and asymmetrical synaptic inputs from voltage-clamp recordings (Fig. 5C). The bistratified dendritic arbor (Fig. 5D–F) tightly costratified with cholinergic bands (Fig. 5G) in the IPL. Sixteen DSGCs were recorded from seven Epb treated retinas, the population data are shown in Fig. 3. These experiments showed that synchronized activities mediated by ACh are not essential for the development of retinal DS circuitry.

### Spiking activity is not required for the development of DS circuitry

In Epb treated retina, the activity of about half of the RGCs was completely silenced, but for the remainders, the spontaneous activity was significantly increased [26], [29], [42]. Because spiking activity has been shown to be sufficient to induce retino-geniculate lamination [31], we further examined if spiking activity is sufficient to induce formation of the DS circuitry. We performed daily intravitreous injection of a voltage gated sodium channel blocker, tetrodotoxin (TTX) [30], [43]. The sustained effectiveness of injected TTX was verified using same experiments as for BMI and Epb (Fig. S2E, H, I).

**Figure 6 pone-0019477-g006:**
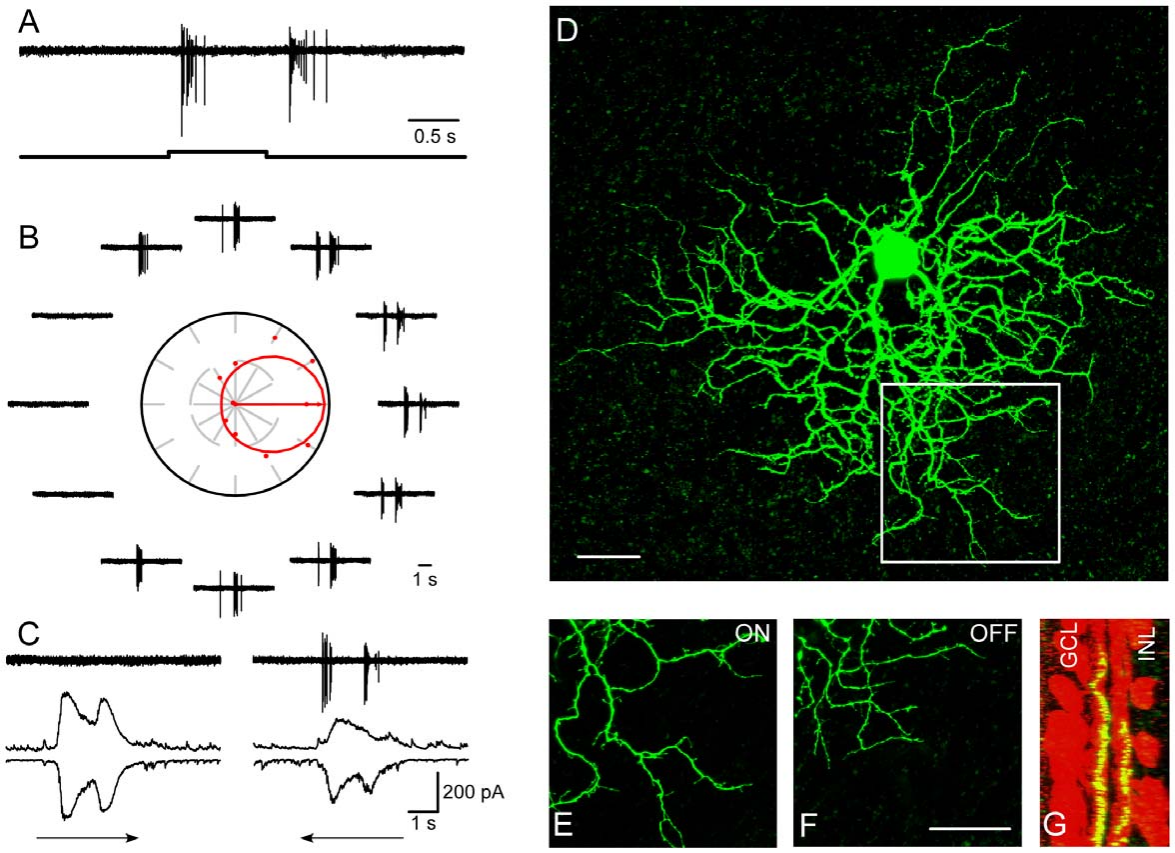
Normal DS in TTX treated retina. A: Clear ON and OFF responses to a flashing spot. B: Responses to a moving rectangle exhibiting strong directional selectivity. C: Voltage clamp responses to null and preferred direction motion. Membrane potential was held at −65 mV and 0 mV, respectively. D: Dendritic arbor of the recorded cell. E–G: The region indicated by the white square in D. E and F: Dendrites in the ON and OFF sublaminae. G: The side view showing clear costratification with cholinergic bands. Scale bar: 30 µm.

Almost normal DS responses can still be recorded from retinas receiving daily TTX injections. Fifteen DSGCs were recorded from eleven TTX treated retinas (Fig. 3) and detailed physiological and morphological data are shown in Figure 6 (A–G). These experiments demonstrated that spiking activity in both SACs and RGCs are not critical for the formation of retinal DS circuitry.

### Normal development of DS circuitry without cholinergic or spiking activity

**Figure 7:**
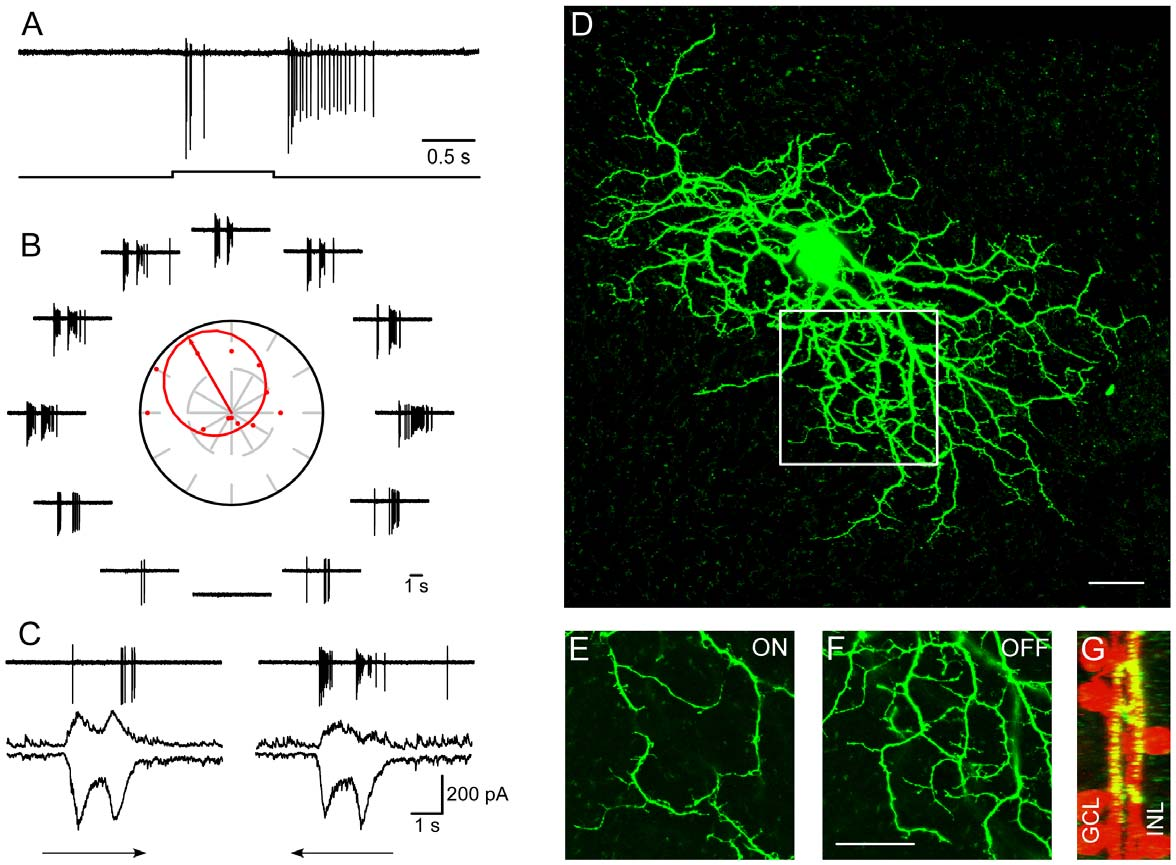
Normal DS in the retina treated by TTX plus Epb. A: ON and OFF responses were elicited by a flashing spot. B: Responses to a rectangle moving in 12 directions. Clear direction selectivity could be observed. C: Asymmetric input evoked by motion from null and preferred directions. Membrane potential was held at −65 mV and 0 mV, respectively. D: Dendritic morphology of the recorded cell. E–G: The region indicated by the white square in D. The dendrites in the ON and OFF sublaminae are shown in E and F. G: The side view showing costratification of DSGC dendrites and cholinergic bands. Scale bar: 30 µm.

Calcium imaging experiments showed that the ACh induced synchronized activities still existed in the TTX treated early postnatal retinas [44], in forms of synchronized elevation in intracellular calcium among neighboring cells. To completely rule out the involvement of any possible synchronized synaptic activity and spiking activity, we performed intraocular injection of TTX and Epb simultaneously. Normal DS responses were observed in 15 cells from 13 retinas (Fig. 3). Detailed physiological and morphological data are shown in Figure 7 (A–G). These experiments showed neither ACh driven synaptic activities nor synchronous or spontaneous spiking activities are required for the development of retinal DS circuitry.

The homeostatic properties of DSGCs are not changed in retinas treated by Epb and TTX (Fig. 4A–E), very similar to the results observed in BMI experiments.

## Discussion

The present study is somewhat surprising because experience or activity dependent plasticity is involved in formation of neural connections in many sites of the visual system, such as the morphological modification of the neurons in the rat retina [45], [46], the formation of eye specific lamination in the lateral geniculate nucleus and precise mapping of geniculocortical connections [25], [26], [27], [28], [29], [30]. Experience is also critical in development of direction selectivity in visual cortical neurons [47].

On the other hand, it is not surprising because at the earliest stage of another sensory system, the olfactory system [48], the wiring of axon terminals to specific glomeruli is controlled by the type of olfactory receptor expressed in the olfactory neurons [49], [50], [51], although there is increasing evidence showing activity also plays a role in regulating the axon terminal targeting [52], [53], [54].

Most recent evidence showing two subtypes of ON-OFF DSGCs can be genetically labeled very early in development [55], [56] suggested these cells are destined to code a particular motion direction at the time or very soon after they are differentiated. However, the specification of cell type does not necessarily mean the completion of neural circuitry. In fact, several pieces of evidence suggest the formation of directional circuitry takes place in the first two postnatal weeks. When the morphology of RGCs were surveyed early in development, very few cells exhibited adult-like dendritic morphology [57], [58], also the relationship between SACs and bistratified RGCs became increasing closer after P3 [59]. Most recently, direct evidence showing asymmetrical inhibition from SACs to DSGCs emerges after P7 [23].

Two recent studies reported that the postsynaptic GABAergic current in DSGCs induced by activating SACs is still symmetric at P7, but becomes asymmetric at P14 and older mice retinas [23]. Using optogenetic approach, Yonehara and colleagues demonstrated the asymmetrical inhibition between SACs and DSGCs is established around P8 [60]. With dual patch clamp recordings, the synaptic strength between null-side SACs and DSGC has been observed to significantly increase compared with the prefer-side during the second postnatal week [23], indicating a selective strengthening of inhibitory synapses between SACs and DSGCs. These findings are consistent with the previous report that DS responses emerge at the onset of light sensitivity at P11 [12].

Bipolar cell terminals reach the inner plexiform layer at P6, and the expression of vesicular glutamate transporter 1 (VGluT1) can be detected at P8 [61] coinciding with the establishment of the asymmetrical inhibition from SACs to DSGCs [60]. To address the role of spontaneous retinal activity during the establishment of DS circuitry further, Wei et al. injected muscimol or gabazine to activate or block GABAergic activity and showed that no change was observed in the asymmetric connection between SACs and DSGCs [23]. Our results ruled out the involvement of not only GABAergic, but also the cholinergic and Na^+^ dependent action potential activities in the development of retinal DS circuitry. Furthermore, our results showed the GABA activity was not necessary for the initiation of contacts between SACs and DSGCs. Combined with previous findings ruling out light driven activities [11], [12], [13], the present study strongly suggests a genetic mechanism in the formation of selective connections between SACs and DSGCs.

## Materials and Methods

### Ethical approval

Use and handling of animals were strictly in accordance to the institutional guidelines and the Society for Neuroscience's policies on the use of animals and human subjects in neuroscience research [Approval Number: SYXK (SPF) 2007-119]. Animals were naturally mated and pups grew up with their mothers until the experimental day. They were maintained on a 12 hours dark/light cycle and the mothers were housed with libitum food (rat chow) and water (tap water). All pups were anesthetized and sacrificed under the guidelines mentioned above. And all efforts were made to minimize suffering.

### Animals

Adult and new born SD rats were obtained from Charles River Laboratories and used in the experiments. Data were obtained from fifty nine early postnatal rats.

### Intraocular Injections

We adopted procedures previously described for intraocular injection [25], [30]. Before P8, animals were anesthetized with hypothermia and after P8 with ether. BMI (10 mg/mL, Sigma, St. Louis, MO, USA) in saline, or 0.2 mM TTX (Sigma, St. Louis, MO, USA) in 3.5 mM citrate buffer and/or 1 mM Epibatidine (Biomol, Plymouth Meeting, PA, USA) in 0.01 M PBS [26], [39], [43] made to the final volume of 1 µL, was injected daily intravitreally from P0 till the day before experiment (P13 to P18). The procedure was carried out under a dissecting microscope to insure clear visibility of the needle tip and every attempt was made to repeatedly inject through the same puncture in the sclera.

In the drug verification experiment, the eyeball received intraocular injection 24 h ago was enucleated and put in a small chamber. We collected as much vitreous humor as possible with a pipette after removing the cornea and lens, and applied the vitreous extracts onto the retina in an oxygenated recording chamber containing about 1 mL Ames medium.

### Visualization of retino-geniculate projection

Cholera toxin B-Alexa Fluor 488 (Molecular Probes, Eugene, OR, USA, 2 µL of 0.5 mg/mL CTB in 0.01 M PBS) was injected into the treated eye and cholera toxin B-Alexa Fluor 594 ((Molecular Probes, Eugene, OR, USA) was injected into the intact eye [26]. Animals were sacrificed 3 days later and intracardially perfused with 4% paraformaldehyde (PFA) in 0.1 M PB. The brains were postfixed overnight in 4% PFA, cryoprotected in 30% sucrose solution (0.01 M PBS) and embedded in OCT. Coronal sections were cut at 50 µm on a cryostat (Leica CM1950). Twenty four sections were usually obtained throughout the LGN, and illustrations were selected from 9 to 16^th^ section. For each selected section, 10 images were captured in 30 µm depth. Areas of both the dLGN and the ipsilateral projection (both the treated and intact sides) were calculated for every single image. Images were captured using a confocal microscope (Olympus FluoView™ FV500). Brightness and contrast were adjusted using Image J 1.42 (National Institutes of Health) and Adobe Photoshop CS2 (Adobe Systensm Inc., San Jose, CA, USA).

### Patch clamp recordings and data analysis

The procedures of patch clamp recording and data analysis have been described [4]. Briefly, rats were dark adapted for at least 2 hours then sacrificed with overdosed Sumianxin (mixture of xylazole, haloperidol and dihydroetorphine, 5 mL/kg), obtained from Institute of Veterinary Sciences, Academy of Military Medical Sciences, (Changchun, China). Eyes were enucleated immediately and a small cut was made at the temporal position (in order to orient the retina). Retinas were isolated and attached on black Millipore filters (AABP02500, Millipore, Billerica, MA, USA) with ganglion cell side up. Then the retinas were transferred into a recording chamber, continuously perfused with oxygenated bicarbonate-buffered Ames medium at 35°C at a rate of 5 mL/min.

After removing the inner limiting membrane with a pipette (filled with Ames solution), somas were exposed and RGCs had a medium sized elliptical soma were targeted, loose patch recording were carried out to identify ON-OFF DSGCs.

The closest stimulating direction to the vector sum of total spiking responses in 12 directions has been defined as preferred direction, and the direction selective index (DSI) is calculated as
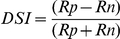
where 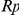 refers to responses in preferred direction and 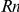 indicates the responses in the opposite direction. While the tuning curves of responses to motion stimuli (red circles in Fig 2B, 5–7B and S1B) are fit with the von Mises distribution:
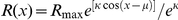
Where 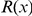 is the response to the stimulus moving in a given direction 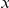 in radians, 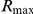 is the maximum response, 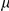 is the preferred direction in radians, and 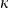 is the concentration parameter used to calculate tuning width. The tuning width is estimated as the full width at half height of the von Mises fit:
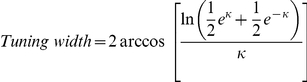
DSI [4] and tuning width [13], [62] were used to evaluate the directional preference of the DSGC and only cells with DSI >0.3 were further investigated.

The voltage clamp recordings were carried out with a pipette filled with intracellular solution (mM: 120 caesium methanesulphonate, 0.5 CaCl_2_, 5 EGTA, 10 Hepes, 4 ATP, 0.5 GTP, and 5 QX-314, adjusted to pH 7.2 with 1 M CsOH) mixed with 0.5% neurobiotin (Molecular Probes, Eugene, OR, USA) and 0.1% Lucifer Yellow (Sigma, St. Louis, MO, USA). The impedance of the pipette is 8∼12 MΩ.

Data were collected with Axonpatch 200B amplifier, Digidata 1322A and pClamp 8.2 (Molecular Devices, Sunnyvale, CA USA). Offline data analysis was carried out using MATLAB 7.7 (The MathWorks, Inc. USA) and OriginPro 8.0 (OriginLab Corp., Northampton, MA, USA).

### Visual stimulus

Light stimuli about 0.35×10^11^ photons cm^−2^ s^−1^ was displayed on a monitor (Sony E230) and focused on the retina through microscope condenser. Four types of light stimuli were generated: (1) a 150 µm (in diameter) spot flashing for 1 s was used to determine the response polarity and receptive field, (2) a 100×500 µm rectangular moving parallel to its long axis at 19 deg s^−1^ over 1500 µm in 12 directions at 30° intervals was used to identify the directional preference, (3) flashing light spot of various diameter (from 50 to 800 µm, lasting for 1 s, repeating 3 times for each diameter) was used to check the center-surround interaction, and (4) moving square wave gratings (bright and dark, both are 200 µm width, 1 Hz) with different contrasts (from 0% to 100%) were used to detect the homeostasis of DSGCs. Percent contrast was defined as (L_max_−L_min_)/(L_max_+L_min_), where L_max_ and L_min_ were the maximum and minimum of luminance in this stimuli pattern.

## Supporting Information

Figure S1 **A DSGC in the rat retina.** A: Transient ON and OFF responses to a flashing spot. B: Responses to a rectangle moving in 12 directions, both the polar plot and spike traces show strong directional selectivity. C: Excitatory and inhibitory synaptic inputs when the membrane potential was held at −65 mV and 0 mV, respectively. D: Dendritic morphology of the recorded cell. E–G: The region indicated by the white square in D. The dendritic stratification in the ON and OFF sublaminae is shown in E and F, and the side view illustrated in G, showing costratification with ChAT bands. H: Spike traces and polar plot of a P13 DSGC. I: Dendritic morphology of the recorded P13 DSGC. Scale bar: 30 µm.
(TIF)

Figure S2 **Effectiveness of intravitreous injection of epibatidine and TTX.** A, B: Retino-geniculate projection in animal treated by daily citrate buffer monocular injection. C, D: Retino-geniculate projection is severely disrupted visualized by intraocular injection of CTB, the ipsilateral projection of the uninjected eye (red) is much expanded (C), and the ipsilateral projection of the injected eye clearly reduced (D). Scale bar: 200 µm. E: Comparison of ipsilateral projection between intact and treated eye. N = 10, Data were analyzed using one way ANOVA, and presented in AVG±SE. *: p<0.05. F: Vitreous extract from an eye injected with Epb 24 hours earlier completely blocked synchronous bursting from a pair of neighboring ganglion cells from a P7 retina, showing sufficiently long lasting effectiveness of Epb. G: Bath application of 40 nM epibatidine blocked synchronous bursting of a pair of neighboring RGCs in a retina receiving 9 consecutive intraocular injections from birth, showing no tolerance to repeated Epb injection. H: Vitreous humor from an eye treated with TTX 24 hours before completely blocked action potential. I: Bath application of 200 nM TTX blocked action potential of an RGC from a retina receiving 17 days of consecutive TTX injection.
(TIF)
