# Supplementary figures and images for: Direction-Selective Circuitry in Rat Retina Develops Independently of GABAergic, Cholinergic and Action Potential Activity
Source: PLoS One. 2011 May 5;6(5):e19477. doi: 10.1371/journal.pone.0019477 (PMC3088673; doi:10.1371/journal.pone.0019477)

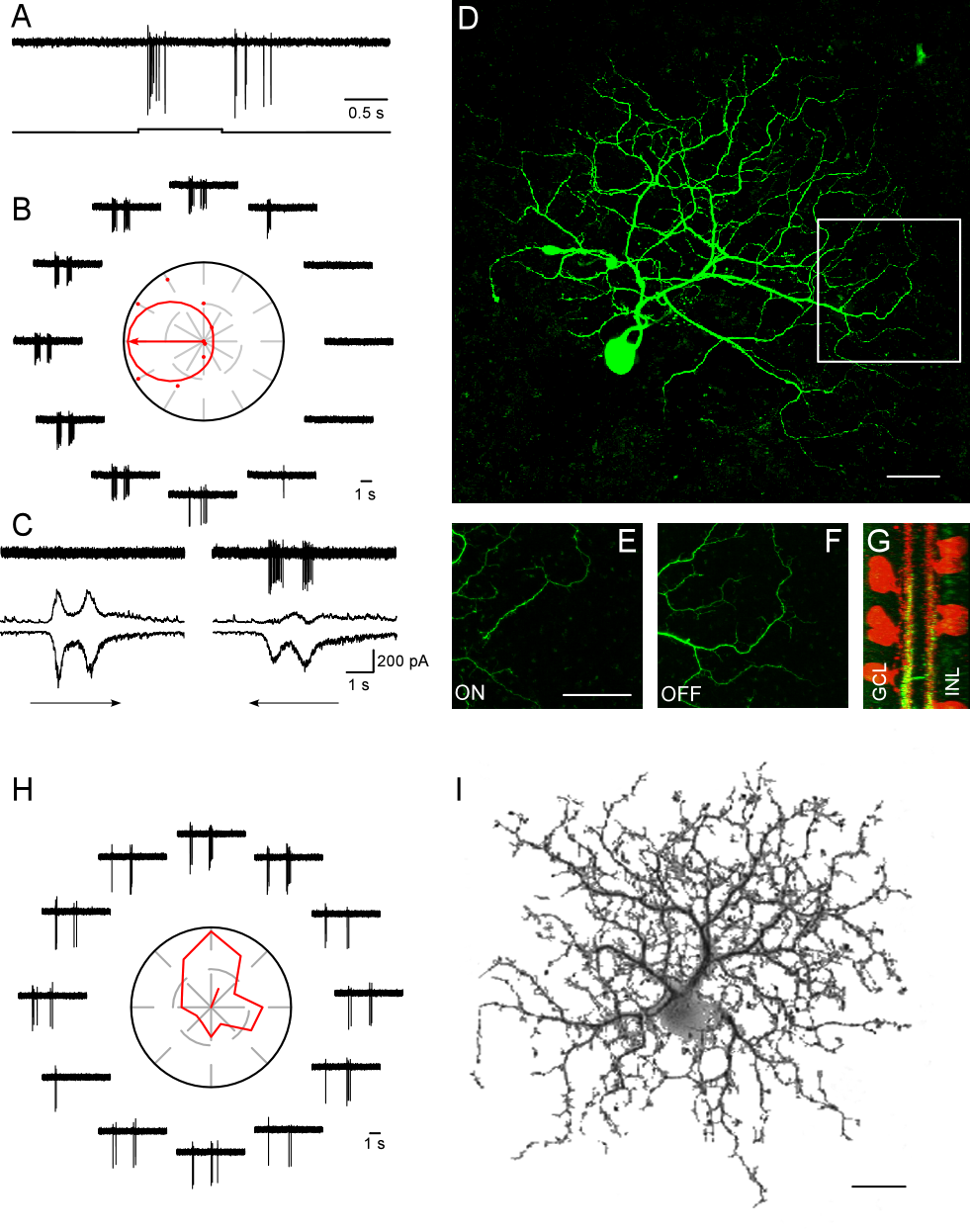

Supplement: Figure S1 — A DSGC in the rat retina. A: Transient ON and OFF responses to a flashing spot. B: Responses to a rectangle moving in 12 directions, both the polar plot and spike traces show strong directional selectivity. C: Excitatory and inhibitory synaptic inputs when the membrane potential was held at −65 mV and 0 mV, respectively. D: Dendritic morphology of the recorded cell. E–G: The region indicated by the white square in D. The dendritic stratification in the ON and OFF sublaminae is shown in E and F, and the side view illustrated in G, showing costratification with ChAT bands. H: Spike traces and polar plot of a P13 DSGC. I: Dendritic morphology of the recorded P13 DSGC. Scale bar: 30 µm. (TIF) [file pone.0019477.s001.tif]

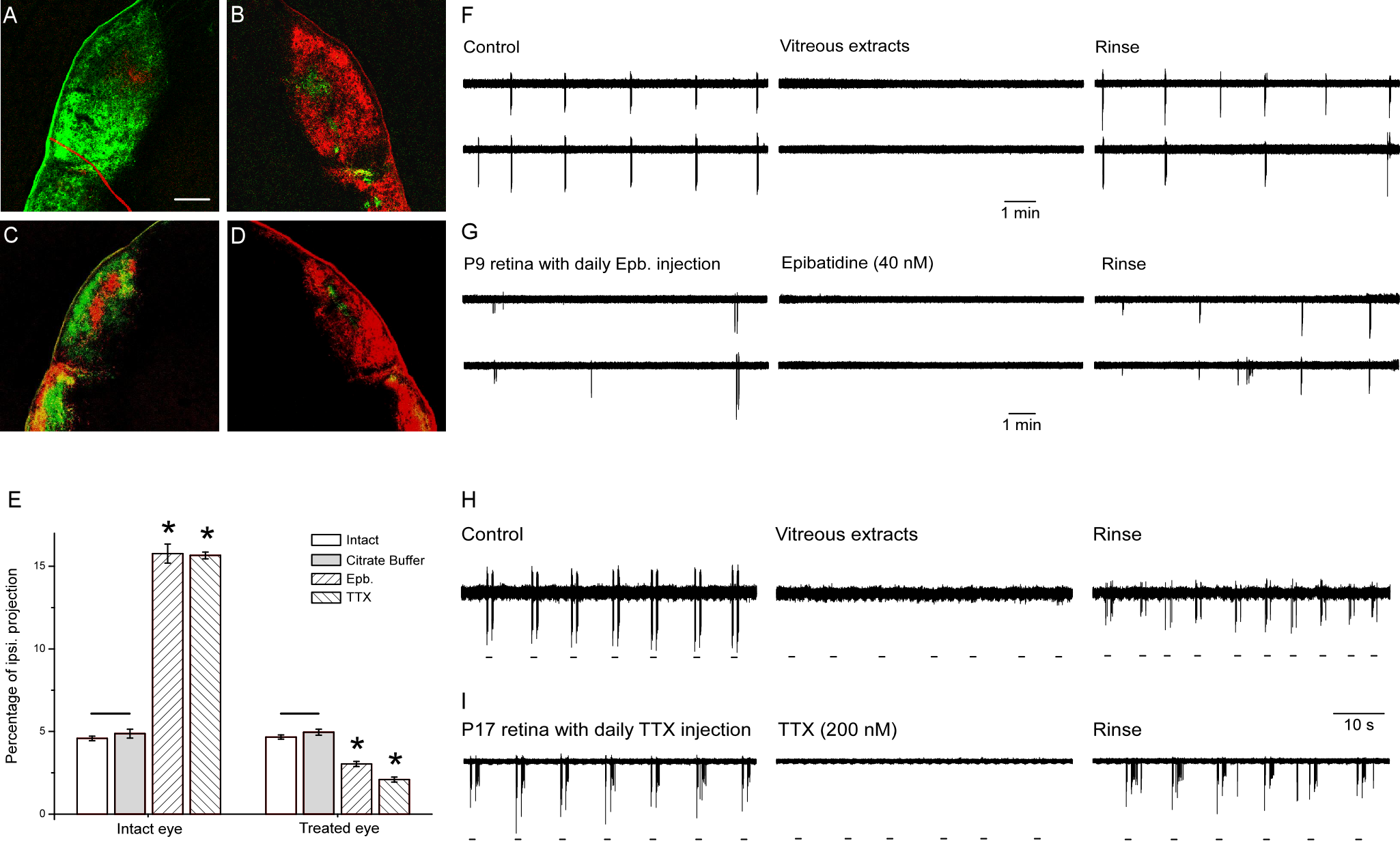

Supplement: Figure S2 — Effectiveness of intravitreous injection of epibatidine and TTX. A, B: Retino-geniculate projection in animal treated by daily citrate buffer monocular injection. C, D: Retino-geniculate projection is severely disrupted visualized by intraocular injection of CTB, the ipsilateral projection of the uninjected eye (red) is much expanded (C), and the ipsilateral projection of the injected eye clearly reduced (D). Scale bar: 200 µm. E: Comparison of ipsilateral projection between intact and treated eye. N = 10, Data were analyzed using one way ANOVA, and presented in AVG±SE. *: p<0.05. F: Vitreous extract from an eye injected with Epb 24 hours earlier completely blocked synchronous bursting from a pair of neighboring ganglion cells from a P7 retina, showing sufficiently long lasting effectiveness of Epb. G: Bath application of 40 nM epibatidine blocked synchronous bursting of a pair of neighboring RGCs in a retina receiving 9 consecutive intraocular injections from birth, showing no tolerance to repeated Epb injection. H: Vitreous humor from an eye treated with TTX 24 hours before completely blocked action potential. I: Bath application of 200 nM TTX blocked action potential of an RGC from a retina receiving 17 days of consecutive TTX injection. (TIF) [file pone.0019477.s002.tif]
